# Comparing self-reported medication adherence measures with hair antiretroviral concentration among people living with HIV in Guangxi, China

**DOI:** 10.1186/s12981-020-00265-4

**Published:** 2020-03-02

**Authors:** Quan Zhang, Xiaoming Li, Shan Qiao, Zhiyong Shen, Yuejiao Zhou

**Affiliations:** 1 South Carolina SmartState Center for Healthcare Quality (CHQ), Arnold School of Public Health, University of South Carolina, Discovery I, 915 Greene Street, Columbia, SC 29028 USA; 2 Institute of Pedagogy and Applied Psychology, School of Public Administration, Hohai University, Nanjing, Jiangsu China; 3 Guangxi Zhuang Autonomous Region Center for Disease Control and Prevention, Nanning, Guangxi China

**Keywords:** Antiretroviral adherence, Self-report, Hair concentration, Tenofovir, People living with HIV

## Abstract

**Background:**

Antiretroviral adherence is essential to HIV treatment efficacy. Various self-reported measures are commonly used for assessing antiretroviral adherence. Limited data are available regarding the validity of those self-reported measures in comparison with long-term objective biomarkers of adherence measures such as hair measures.

**Methods:**

Self-reported adherence (frequency, percentage, and visual analog scale [VAS]) and hair tenofovir concentration were evaluated at a single time point from 268 people living with HIV in China. The responses to each of three self-reported measures were converted into percentage and then dichotomized as “optimal” (100%) vs. “suboptimal” (less than 100%) adherence. Two composite adherence scores (CAS) were created from the three self-reported measures: (1) an overall adherence was the average percentage of the three self-reported measures; (2) responses were termed optimal adherence if participants reporting optimal adherence in all three self-reported measures, while were termed suboptimal adherence. Hair tenofovir concentration was also dichotomized as “optimal” (above the limit of quantitation, 36 pg/mg) vs. “suboptimal” adherence (blow 36 pg/mg). Spearman correlation, kappa statistics, and logistic regression analysis were used to calculate the correlations, agreements, and predictions of self-reported measures with hair measure, respectively.

**Results:**

Overall adherence, but any of the three self-reported adherence, was correlated with hair tenofovir concentration (*r *= 0.13, *p *< 0.05). Self-reported optimal adherence in VAS and CAS measures were agreed with and predicted optimal adherence assessed by hair measure (Kappa = 0.107, adjusted OR = 1.88, 95% CI 1.03–3.45; Kappa = 0.109, adjusted OR = 1.80, 95% CI 1.02–3.18; all *p *< 0.05, respectively).

**Conclusion:**

VAS may be a good individual self-reported measure for antiretroviral adherence, and CAS may be a good composite self-reported measure for antiretroviral adherence.

## Background

In China, antiretroviral therapy (ART) has been available for HIV treatment at no cost to people living with HIV (PLHIV) for more than a decade. However, despite the increased availability of antiretroviral medications, HIV-related morbidity, mortality, and new infection rate continually increased over the years in China from 2004 to 2016 [1]. Both clinical trials and cohort studies have shown that the effectiveness of antiretroviral medications is highly dependent on adherence [2–4]. Suboptimal adherence severely undercuts antiretroviral effectiveness and places PLHIV at risk for virologic failure, increases the risk of onward HIV transmission, and limits the impact of HIV treatment resources [5, 6]. Detecting and addressing suboptimal adherence to antiretroviral is an essential component of HIV treatment and management in China and other countries. Consequently, one of the challenges is how to assess antiretroviral adherence accurately.

Currently, antiretroviral adherence measures vary widely, and there is still no single “gold standard” for accurate measurement of adherence [7, 8]. With the desires for objective and accurate measures, researchers employed medication electronic drug monitoring [9], pharmacodynamics responses (e.g., viral load) [10], or antiretroviral concentration in various pharmacokinetic metrics such as plasma [11], saliva [12], urine [13], dried blood spots [14], and hair [15]. However, these measures are often impractical in resource-limited settings because of the high cost and logistic complexities [7, 8, 16]. Alternatively, researchers have employed various self-reported measures that differ in content (e.g., pill taken or pill missed), response option (e.g., percentage or rating), and recall timeframe (e.g., 3 days or 30 days) to estimate adherence [17–20]. Self-report remains the most widely and frequently used method in measuring antiretroviral adherence because of its low-cost, noninvasiveness, minimal patient burden, and ease of administration [17, 21]. However, because of possible biases inherent in self-reporting (e.g., social desirability, memory decay, inaccuracy in estimation, and error of recall) [22, 23], examining the validity of various self-reported measures has been a focus of recent research.

Several studies have examined the concurrent validity of various self-reported measures with other device-based measures [17] or biological measures (e.g., viral load, and antiretroviral concentration in various pharmacokinetic metrics) [10, 19, 24–26]. While these studies provide important information regarding the validity of self-reported measures, data are limited in Chinese PLHIV and comparison of those self-reported measures with long-term biological measures of adherence. In addition, some studies used a composite adherence score (CAS) from several self-reported measures with the hope to offset or reduce potential biases in self-reporting [27–29]. Several studies employed CAS measures to predict objective measures of adherence (e.g., device-based measures and viral load) [17, 18, 26], and did not find advantages over the individual self-reported measures. However, it is unclear whether CAS will offer advantages over the individual self-reported measures in predicting biological measures of adherence that reflects long-term antiretroviral exposure.

Given antiretroviral concentration in hair reflects uptake from the systemic circulation over an extended time window and human hair grows at an average rate of 1 cm per month, antiretroviral concentration in 1 cm hair approximately represents a 1-month window of antiretroviral exposure. Therefore, Hair antiretroviral concentration has been considered as a long-term adherence biomarker [30, 31]. One of the most commonly used antiretroviral components is tenofovir disoproxil fumarate (TDF) which has been provided at no-cost to PLHIV in China by the Chinese government since 2012. Hair tenofovir concentration has been used as a long-term adherence biomarker among PLHIV [32] and populations at risk for HIV infection [33–35] in the US and Africa. More importantly, Liu et al. directly observed a strong linear relationship between the frequency of TDF dosing and hair tenofovir concentration in healthy volunteers that could provide a valid standard for comparing self-reported measures [36].

Therefore, the present study was designed to compare three common self-reported measures and their CAS with hair tenofovir concentration among PLHIV received TDF-based ART in China. We plan to examine the relationships between self-reported adherence and hair tenofovir concentration at a single study visit.

## Methods

### Study participants

Data used in the current study were collected from September 2016 to January 2017 as part of an HIV disclosure study among 446 PLHIV in Guangxi, China [37]. With the assistance and collaboration of Guangxi Center for Disease Prevention and Control (Guangxi CDC), we randomly selected 10 sites with the largest number of HIV/AIDS cases from 17 cities and 75 counties in Guangxi. With the referral from medical staff or HIV case managers at the study sites, local team members screened PLHIV for eligibility and discussed the benefits and risks of the study and invited them to join. After obtaining written informed consent, participants completed the survey one-on-one with an interviewer in private rooms of the clinics. The interviewers were medical staff or HIV case managers in the HIV clinics who had received intensive training on research ethics and interview skills with PLHIV prior to the field data and hair specimen collection.

### Hair collection and assay

Approximately 150 strands of hair were cut from the posterior vertex region as close as possible to the scalp following a standard protocol [38, 39] after participants finished the survey. The proximal section of the hair sample (about 1 cm, reflecting the last month of TDF dosing) was rinsed with methanol and dried under a blow of pure nitrogen gas. Then hair sample was chopped to 1–2 mm length segments with scissors, and 10 mg weighed, processed, and analyzed using high-performance liquid chromatography (Agilent 1200 HPLC system, Agilent, Waldbronn, Germany) and tandem mass spectrometry (ABI 3200Qtrap, ABI, Foster City, CA, USA) (LC–MS/MS) [40, 41]. Briefly, following the 2013 FDA guideline, The tenofovir in the cut hair sample was extracted with methanol and internal standard in a 37 °C shaking water bath overnight (> 16 h) and then analyzed by an LC–MS/MS [40]. Standard curves were linear in the range of 36–1250 mg with good linearity (R^2^ = 0.994) and reproducibility. The relative error (%) and precision [coefficients of variation (CV)] for spiked quality control hair samples at low, medium and high concentrations were all < 12%. The recoveries at low, medium and high concentrations were all ≥ 98.2%. The limit of quantitation (LOQ) was 36 pg/mg. No significant matrix ionization suppression was observed.

Based on two previous studies, adherence was defined as detectable antiretroviral in dried blood spots [24], and the range of hair tenofovir concentration was 21 to 53 pg/mg when the subject took TDF 7 days/week (100% adherence) [36], we defined hair measure of optimal adherence as hair tenofovir concentration above LOQ, and suboptimal adherence as hair tenofovir concentration below LOQ.

### Self-reported measures

#### Frequency of adherence behavior

The participants were asked the overall frequency of their adherence behavior via the question: “In last month, how often did you take all your HIV medications as your doctor prescribed them?” with a 5-point response option (none of the time, few of the time, some of the time, most of the time, all of the time) [17]. The responses to frequency measure were quantified in 25% increments (e.g., none of the time = 0%, few of the time = 25%, some of the time = 50%, most of the time = 75%, and all of the time = 100%) [19].

#### Percent of days of adherence

The participants were asked the question: “In last month, how many days were you able to take your medications exactly as prescribed?”. The percent of days of adherence was calculated by dividing the reported number of days by 30 days and then converted into a percentage [10].

#### Visual analog scale

A modified visual analog scale (VAS) [21] for missing doses was employed with the following instruction: “Place a mark (X) at the point on the line that shows your best guess about how many of you prescribed medications you have missed in last month.” The 11 possible responses to the scale ranged from “0” representing no missing doses, “5” representing missing half doses, to “10” representing missing all doses. The responses to VAS were appropriately converted into percent of adherence in 10% increments (e.g., “0” was assigned 100%, and “10” was assigned 0%) with a higher percentage indicating a better adherence behavior [17].

#### Composite adherence scores

Given that self-reported data are generally skewed and overestimated, the responses to each of three self-reported measures were converted into percentage and then dichotomized as “optimal” (100%) vs. “suboptimal” (less than 100%) adherence. Two CAS were created from the three self-reported measures: (1) an overall adherence was the average percentage of the three self-reported measures; (2) responses were termed optimal adherence if participants reporting optimal adherence in all three self-reported measures, while were termed suboptimal adherence.

### Covariates

Participants provided information on their socio-demographic and HIV-related characteristics including age, sex, ethnicity, marital status, work status, education experience, monthly household income, ART regimen, duration of HIV diagnosis, and duration of TDF-based ART. The duration of HIV diagnosis and TDF-based ART referred to the time period from the initial date of confirmed HIV diagnosis and the start date of TDF-based ART to the date of survey, respectively.

### Statistical analysis

The self-reported adherence was treated as both continuous and dichotomous (Optimal vs. suboptimal adherence) in the analysis. Likewise, hair tenofovir concentration was also treated as both continuous and dichotomous (Optimal vs. suboptimal adherence). Descriptive statistics were used to summarize the sample characteristics, self-reported adherence, and hair tenofovir concentration. Spearman correlation was used to calculate the correlations of continuous self-reported measures with continuous hair measure. Kappa statistics were used to calculate the agreements of self-reported measures with hair measure, respectively. Univariate logistic regression analysis was used to calculate the predictions of self-reported measures of optimal adherence with hair measure of optimal adherence. Multivariate logistic regression analysis was conducted when the covariates were included in the model. SPSS 26 was used for all statistical analyses.

## Results

Of 10 clinics selected, 293 PLHIV receiving TDF-based ART were identified, of which 25 were excluded because of the insufficient quantity of hair samples for assaying (less than 10 mg, n = 23) or lack of self-reported measures (n = 2), leaving an effective sample of 268 PLHIV with a mean (SD) age of 42 (8) years. Of the participants (Table 1), 69.4% were men, 70.1% were of Han ethnicity, 79.5% were married, 77.2% were full or part-time employed, and 93.7% were on TDF-based first-line ART. Less than half (47.8%) of participants completed no more than elementary education. Most of the sample had low income with 73.9% reporting a monthly household income of less than 3000 Chinese Yuan (or approximately US$460 during the time of the survey). The median duration of HIV diagnosis was 17.5 months and the median duration of TDF-based ART was 16.5 months.

**Table 1:** Characteristics of the study sample

Characteristics	All samples	Male	Female
n (%)	268 (100%)	185 (69.4%)	83 (30.6%)
Age (M ± SD), years	41.9 ± 7.8	42.1 ± 7.5	41.6 ± 8.36
Ethnicity, n (%)			
Han	188 (70.1%)	135 (73.0%)	53 (63.9%)
Others	80 (29.9)	50 (27.0%)	30 (36.1%)
Education, n (%)			
Elementary school or below	128 (47.8%)	78 (42.2%)	50 (60.2%)
Middle school	113 (44.2%)	87 (47.0%)	26 (31.3%)
High school or higher	27 (10.1%)	20 (10.8%)	7 (8.4%)
Marital status, n (%)			
Single	27 (10.1%)	26 (14.1%)	1 (1.2%)
Married/cohabitating	213 (79.5%)	145 (78.4%)	67 (81.9%)
Others	28 (10.4%)	14 (7.6%)	14 (16.9%)
Work status, n (%)			
Full-time employed	156 (58.2%)	121 (65.4%)	35 (42.2%)
Part-time employed	51 (19.0%)	24 (13.0%)	27 (32.5%)
Unemployed	61 (22.8%)	40 (21.6%)	21 (25.3%)
Income per month, (yuan), n (%)			
< 1000	51 (19.0%)	41 (22.2%)	10 (12.0%)
1000–2999	149 (55.6%)	96 (51.9%)	53 (63.9%)
≥ 3000	68 (25.4%)	48 (25.9%)	20 (24.1%)
ART regimen			
TDF based first-line ART, n (%)	251 (93.7%)	179 (96.8%)	72 (86.7%)
TDF based second-line ART, n (%)	17 (6.3%)	6 (3.2%)	11 (13.3%)
Duration of HIV diagnosis, median (range), months	17.5 (2–37)	17 (2–37)	18 (3–34)
Duration of TDF-based ART, median (range), months	16.5 (1–36)	16 (1–36)	18 (2–34)

*M* mean, *SD* standard deviation, *TDF* tenofovir disoproxil fumarate, *ART* antiretroviral therapy

As showed in Table 2, the mean percentage of adherence was more than 95% for all three self-reported and CAS measures. The mean hair tenofovir concentration was 120.75 pg/mg. Spearman correlation statistics showed that only overall adherence, but any of the three self-reported adherence was correlated with hair tenofovir concentration (*r *= 0.13, *p *< 0.05).

**Table 2:** Correlations among hair and self-report measures

Adherence measures	Value, M ± SD (range)	1	2	3	4	5
1. Frequency, %	95.99 ± 10.83, (25–100)	1				
2. Percent, %	98.18 ± 10.43, (0–100)	0.39**	1			
3. VAS, %	95.19 ± 12.56, (0–100)	0.53**	0.56**	1		
4. Overall adherence, %	96.45 ± 8.45, (27–100)	0.76**	0.57**	0.92**	1	
5. Hair TFV concentration, pg/mg	120.75 ± 290.10, (below LOQ–2978.99)	0.06	0.05	0.12	0.13*	1

*VAS* visual analog scale, *TFV* tenofovir, *LOD* limit of quantitation

*p < 0.05; **p < 0.001

As showed in Table 3, the percentages of self-reported optimal adherence were 85.8%, 97.4%, 76.9%, 73.5% for frequency, percent, VAS, and CAS measure, respectively. The percentage of optimal adherence assessed by hair measure was 46%. Kappa statistics showed that self-reported optimal adherence in VAS and CAS measure, but the other two self-reported measures, agreed with optimal adherence assessed by hair measure (Kappa = 0.107, *p *< 0.05; Kappa = 0.109; both *p* < 0.05, respectively).

**Table 3:** Association between hair measure and self-reported measures of optimal adherence

Adherence measures		Hair TFV concentration		Kappa statistics	Univariate model^a^		Multivariate model^b^	
		Optimal 123 (45.9%)	Suboptimal 145 (54.1%)		cOR (95% CI)	*p*	aOR (95% CI)	*p*
Frequency								
Optimal	230 (85.8%)	109 (88.6%)	121 (83.4%)	0.048	1.54 (0.76, 3.14)	0.229	1.37 (0.66, 2.86)	0.400
Suboptimal	38 (14.2%)	14 (11.4%)	24 (16.6%)		–	–	–	–
Percent								
Optimal	261 (97.4%)	122 (98.4%)	140 (96.6%)	0.017	2.16 (0.41, 11.34)	0.362	2.37 (0.44, 12.95)	0.318
Suboptimal	7 (2.6%)	2 (1.6%)	5 (3.4%)		–	–	–	–
VAS								
Optimal	206 (76.9%)	102 (82.9%)	104 (71.7%)	0.107*	1.91 (1.06, 3.46)	0.032	1.88 (1.03, 3.45)	0.041
Suboptimal	62 (23.1%)	21 (17.1%)	41 (28.3%)		–	–	–	–
CAS								
Optimal	197 (73.5%)	98 (79.7%)	99 (68.3%)	0.109*	1.82 (1.04,3.19)	0.036	1.80 (1.02, 3.18)	0.043
Suboptimal	71 (26.5%)	25 (20.3%)	46 (31.7%)		–	–	–	–

* < 0.05. Data are in numbers and percentages [n (%)], TFV is tenofovir. VAS is visual analog scale. CAS is composite adherence score

^a^Logistic regression; cOR is crude odd ratio; aOR is adjusted odd ratio

^b^Adjusted for age, sex, ethnicity, marital status, work status, education experience, monthly household income, duration of HIV diagnosis, ART regimen and duration of TDF-based ART

Logistic regression analysis showed that self-reported optimal adherence in VAS and CAS measures, but other two self-reported measures, predicted optimal adherence assessed by hair measure in both univariate model (crude OR = 1.91, 95% CI 1.06–3.46; crude OR = 1.82, 95% CI 1.04–3.19; both *p *< 0.05, respectively) and multivariate model (adjusted OR = 1.88, 95% CI 1.03–3.45; adjusted OR = 1.80, 95% CI 1.02–3.18; both *p *< 0.05, respectively).

## Discussion

The present study compared three self-reported measures and their CAS with hair tenofovir concentration among Chinese PLHIV receiving TDF-based therapy. We found that (1) adherence assessed by CAS measure was significantly correlated with hair tenofovir concentration; (2) self-reported optimal adherence in VAS and CAS measures were significantly agreed with optimal adherence assessed by hair measure. (3) Self-reported optimal adherence in VAS and CAS measures were significantly predicted optimal adherence assessed by hair measure.

The finding regarding the weak correlations of adherence assessed by three individual self-reported measures with hair measure was generally in line with previous studies by using tenofovir [35, 42, 43] and other antiretroviral medications [15, 44–47] in hair, plasma [19] and dried blood spots [24, 26] among PLHIV and populations at risk for HIV infection. The finding regarding the weak agreements of adherence assessed by three individual self-reported measures with hair measure was also generally in line with the previous study by using antiretroviral medications in dried blood spots [26]. The inconsistency between subjective self-reported measures and objective hair measure might be one of the major reasons for the weak associations and agreements. For example, Alcaide et al. data showed that over 80% of participants reported optimal adherence in 2 individual self-reported measures, but only 74% of the participants had antiretroviral detected in dried blood spots [24]. Our data showed that over 74% of the participants reported optimal adherence in 3 individual self-reported measures and one CAS measure, but only 46% of the participants achieved hair measure of optimal adherence.

The finding regarding CAS measures provides an opportunity to improve the accuracy of self-reported measures and the power of these measures in predicting long-term antiretroviral exposure. However, our findings were not totally consistent with previous studies in which composite scores did not show clear advantages over individual measures in terms of their associations with device-based measures and viral load. Lu et al. found that the association of device-based measures with overall adherence from three self-reported measures (rating, frequency, and percent) was similar to that with individual self-reported measures [17]. Kagee and Nel also found that the association between viral load (detectable vs. undetectable) and overall composite adherence score of six self-reported measures was similar to the associations of viral load with individual self-reported measures [18]. The main reason for the inconsistency between our study and the previous studies might be the difference in the criterion measure used in the studies. For example, the device-based measure is relatively objective but it is prone to either underestimation (e.g., device nonuse and pocket dosing) or overestimation (e.g., curiosity opening) of the actual adherence behaviors [8]. Viral load is an objective measure but it is not informative of adherence patterns and the development of viremia usually results long after an adherence gap has occurred [7]. On the other hand, hair antiretroviral concentration is based on drug ingestion in the body system and can provide a more accurate measure of adherence [31], and hair antiretroviral concentration used in this study is a relatively gold standard because the previous study provided the valuable information about the relationship between TDF dosages and hair antiretroviral concentration in healthy volunteers [36].

While our study has strengths in the relatively large sample with adherence assessed by three self-reported measures with a 1-month recall timeframe and antiretroviral concentration in 1-cm hair as a valid biomarker of adherence, there are some limitations. First, the current study was based on cross-sectional data, which prevents making causal inferences. Future research should aim to study the predictive value of self-reported adherence for long-term antiretroviral exposure in longitudinal designs. Second, the current study only employed three single-term self-reported measures. Some other commonly used self-reported measures (e.g., multi-item measure and other types of single-term measure) need to be validated in the future. Third, the current study employed two different increments to convert the responses of self-reported measures (e.g., 10% for VAS and 25% for percent) into percent of adherence. Although these two increments are justified based on the responses to specific measures, the different increments may affect the associations between self-reported measures and hair tenofovir concentration. Fourth, we chose to use hair antiretroviral concentration as a single criterion to test the validity of self-reported measures in this study. While hair antiretroviral concentration has been previously shown to be an excellent measure for long-term antiretroviral exposure [7, 48, 49], future research should employ multiple criteria such as objective device-based measures and biological measures (e.g., hair antiretroviral concentration and viral load) to examine the validity of self-reported measures in HIV research and clinic care. Fifth, data were not available in the current study on some important factors (e.g., substance use) that might influence both self-reported measures and hair antiretroviral concentration [50, 51]. Those factors should be considered in future research.

## Conclusion

In summary, our findings indicated that CAS may be a good composite self-reported measure for antiretroviral adherence and VAS measure may be a good individual self-reported measure for antiretroviral adherence. Given the importance of adherence in achieving effective HIV treatment, our and previous data also suggest that objective measures of adherence are required in the future due to weak correlations between self-reported measures and objective measures of adherence. However, in the absence of objective measures of adherence, the use of information from multiple self-reported measures for better assessments of antiretroviral medication adherence in HIV research and clinical care in the future, especially in resource-limited settings where the collection and analysis of more objective measures (e.g., hair antiretroviral concentration or other biomarkers) are challenging or unfeasible.

## Data Availability

Data will not be shared as the IRB has no policy to share the data without prior permission.

## Abbreviations

CAS: Composite adherence score
HIV: Human immunodeficiency virus
LC–MS/MS: Liquid chromatography-tandem mass spectrometry
PLHIV: People living with HIV
TDF: Tenofovir disoproxil fumarate
VAS: Visual analog scale
LOQ: Limit of quantitation
